# Patient and caregiver perspectives on mental health in children and adolescents with chronic kidney disease

**DOI:** 10.1093/ckj/sfaf067

**Published:** 2025-03-08

**Authors:** Luca G Torrisi, Anita van Zwieten, Chandana Guha, Marianne Kerr, Amanda Sluiter, Anastasia Hughes, Jonathan C Craig, Aditi Sinha, Allison Dart, Allison A Eddy, Hui-Kim Yap, Stephen I Alexander, Susan L Furth, Joshua Kausman, Allison Jaure

**Affiliations:** Sydney School of Public Health, The University of Sydney, Sydney, Australia; Centre for Kidney Research, The Children's Hospital at Westmead, Westmead, Australia; Sydney School of Public Health, The University of Sydney, Sydney, Australia; Centre for Kidney Research, The Children's Hospital at Westmead, Westmead, Australia; Sydney School of Public Health, The University of Sydney, Sydney, Australia; Centre for Kidney Research, The Children's Hospital at Westmead, Westmead, Australia; Centre for Kidney Research, The Children's Hospital at Westmead, Westmead, Australia; Sydney School of Public Health, The University of Sydney, Sydney, Australia; Centre for Kidney Research, The Children's Hospital at Westmead, Westmead, Australia; Sydney School of Public Health, The University of Sydney, Sydney, Australia; Centre for Kidney Research, The Children's Hospital at Westmead, Westmead, Australia; College of Medicine and Public Health, Flinders University, Adelaide, South Australia, Australia; Department of Pediatrics, Division of Nephrology, All India Institute of Medical Sciences, New Delhi, India; Department of Pediatrics and Child Health, The Children's Hospital Research Institute of Manitoba, University of Manitoba, Winnipeg, MB, Canada; Department of Pediatrics, University of British Columbia, and BC Children's Hospital Research Institute, Vancouver, BC, Canada; Department of Pediatrics, Yong Loo Lin School of Medicine, National University of Singapore, Singapore, Singapore; Centre for Kidney Research, The Children's Hospital at Westmead, Westmead, Australia; Department of Pediatrics, Perelman School of Medicine at the University of Pennsylvania, Children's Hospital of Philadelphia, Philadelphia, Pennsylvania, USA; Department of Nephrology and Murdoch Children's Research Institute, Royal Children's Hospital, Melbourne, Victoria, Australia; Department of Pediatrics, University of Melbourne, Victoria, Australia; Sydney School of Public Health, The University of Sydney, Sydney, Australia; Centre for Kidney Research, The Children's Hospital at Westmead, Westmead, Australia

**Keywords:** adolescents, children, chronic kidney disease, mental health, patient-centered care

## Abstract

**Rationale & Objective:**

Children and adolescents with chronic kidney disease (CKD) are at risk of depression and other mental health conditions, which can impair quality of life, the capacity for self-management and adherence to treatment, and overall health. This study aimed to describe the perspectives of patients and caregivers on mental health in children and adolescents across all stages of CKD.

**Study Design:**

Qualitative study.

**Settings & Participants:**

A secondary analysis of a consensus, multi-stage and inclusive process designed to establish core outcomes for children with CKD [Standardised Outcomes in Nephrology–Children and Adolescents (SONG-Kids)]. A total of 120 children and 250 caregivers, from 53 countries, who participated in 16 focus groups, two consensus workshops and an international Delphi survey were eligible for inclusion.

**Analytical Approach:**

We conducted a secondary thematic analysis of all qualitative data from the (SONG-Kids).

**Results:**

We identified five themes: struggling with a frail and sick identity (demoralized by a restricted lifestyle, shattered body image, victim of bullying, and descending into loneliness and isolation), worried by ongoing uncertainty about health (confronting own mortality and apprehension awaiting medical results), disappointed by narrowed vocational opportunities (unable to reach academic potential and thwarted career goals), distressed by medical trauma (traumatized by invasive procedures, and unrelenting demands of medication and treatment) and despair without adequate psychological support.

**Limitations:**

The transferability of the findings may be limited as the study was conducted in English.

**Conclusion:**

Children and adolescents with CKD may feel vulnerable, experience fear and anxiety about their prognosis and health, harbour a sense of failure with disappointment, and experience medical trauma. Improving ways to address fears and uncertainty about health, disruption to lifestyle and identity, and medical trauma in children with CKD are needed.

KEY LEARNING POINTS **What was known:**

- Children and adolescents with chronic kidney disease (CKD) are at an increased risk of mental health conditions including depression.
- Mental health problems can impair quality of life, self-management and adherence to treatment, and overall health.

**This study adds:**

- Young people with CKD struggle with a frail and sick identity, facing ongoing uncertainty about their health, feel disappointed by limited study and vocational opportunities, and can be traumatized by invasive medical procedures.
- Caregivers of children with CKD feel helpless about the inadequate psychological support for their children.

**Potential impact:**

- Improved recognition and access to mental health support is needed to help young people with CKD cope with fears and uncertainties about their fragile health, medical traumas, and disruptions to their lifestyle and future vocational opportunities.
- More evidence is needed on interventions such as psychoeducation, cognitive behavioural therapy and health coaching to address mental health challenges.

## INTRODUCTION

Children and adolescents with chronic kidney disease (CKD) commonly experience impaired mental health [1]. Children with CKD have a 30% higher prevalence of depression compared with the general age-matched population, with the highest rates of depression in children receiving dialysis [2–4]. Impaired mental health in children with CKD is associated with an increased risk of non-adherence, graft failure and morbidity and mortality [2, 5].

Mental health challenges in children with CKD are likely to have multifactorial drivers. The diagnosis of CKD as well as the complexity of care and treatment burden can compromise the mental health of young patients because of physiological, cognitive and social changes that occur [6]. Lifestyle restrictions, including having to refrain from certain sports or to adhere to dietary restrictions, the side effects of medications, such as changes in appearances and mood swings attributed to steroids, frequent infections and debilitating symptoms, may all contribute to poor self-esteem and body image, stress, academic disruptions and ultimately poor mental health [3]. Hospitalization, ongoing medical appointments, awaiting medical results and undergoing invasive procedures can also trigger or exacerbate distress and mental health challenges [3, 7].

The perspectives of children and caregivers on mental health in children and adolescents with CKD remain lacking. We aimed to describe the perspectives of children and adolescents, and their caregivers, on the mental health of children and adolescents with CKD. The insights gained in this study may contribute to policy and practice strategies to better support mental health in this population.

## MATERIALS AND METHODS

### Context and sources of data

We conducted a secondary analysis of the data from the Standardised Outcomes in Nephrology (SONG-Kids) initiative [8], which aimed to identify critically important core outcomes for trials in children with CKD. Mental health outcomes including depression and anxiety/stress were identified as important in the SONG-Kids initiative [9]. Secondary analysis is a research approach in which pre-existing data are used to verify findings of the previous research or investigate new questions [10]. We extracted and analysed the responses of 120 patients (aged 8–21 years) and 250 caregivers of children with CKD from 53 countries, who participated in 16 focus groups [9], two consensus workshops [9] and an international Delphi survey [11] for the SONG-Kids initiative. The questions used in the SONG-Kids studies were designed to identify outcomes of importance to patients, caregivers and health professionals, and the reasons for their priorities [11, 12]. This included perspectives of patients and caregivers on mental health (which was one of the outcomes included). The Institutional Review Boards that provided ethics approval for the SONG-Kids initiative are listed in Supplementary data, File S1.

We included data on mental health, which was defined by the World Health Organization as a ‘state of mental well-being that enables people to cope with the stresses of life, realize their abilities, learn well, work well, and contribute to their community’ [13]. This included mental disorders, classified as any ‘clinically significant disturbance in an individual's cognition, emotional regulation, or behaviors’ [14].

### Data extraction and analysis

Participant quotations were extracted from all data related to the original transcripts of the SONG-Kids focus groups [8], free-text responses from the Delphi Survey [15] and two consensus workshops [16]. Using HyperRESEARCH version 4.5.6 (ResearchWare, Randolph, MA, USA), the transcripts were coded line-by-line by L.G.T., who inductively identified preliminary themes and sub-themes based on data identified as related to the topic of mental health in the dataset. L.G.T. discussed the analysis with A.J., A.v.Z. and C.G. to ensure that the findings captured the full range and depth of data related to mental health in children with CKD.

## RESULTS

**Figure 1:**
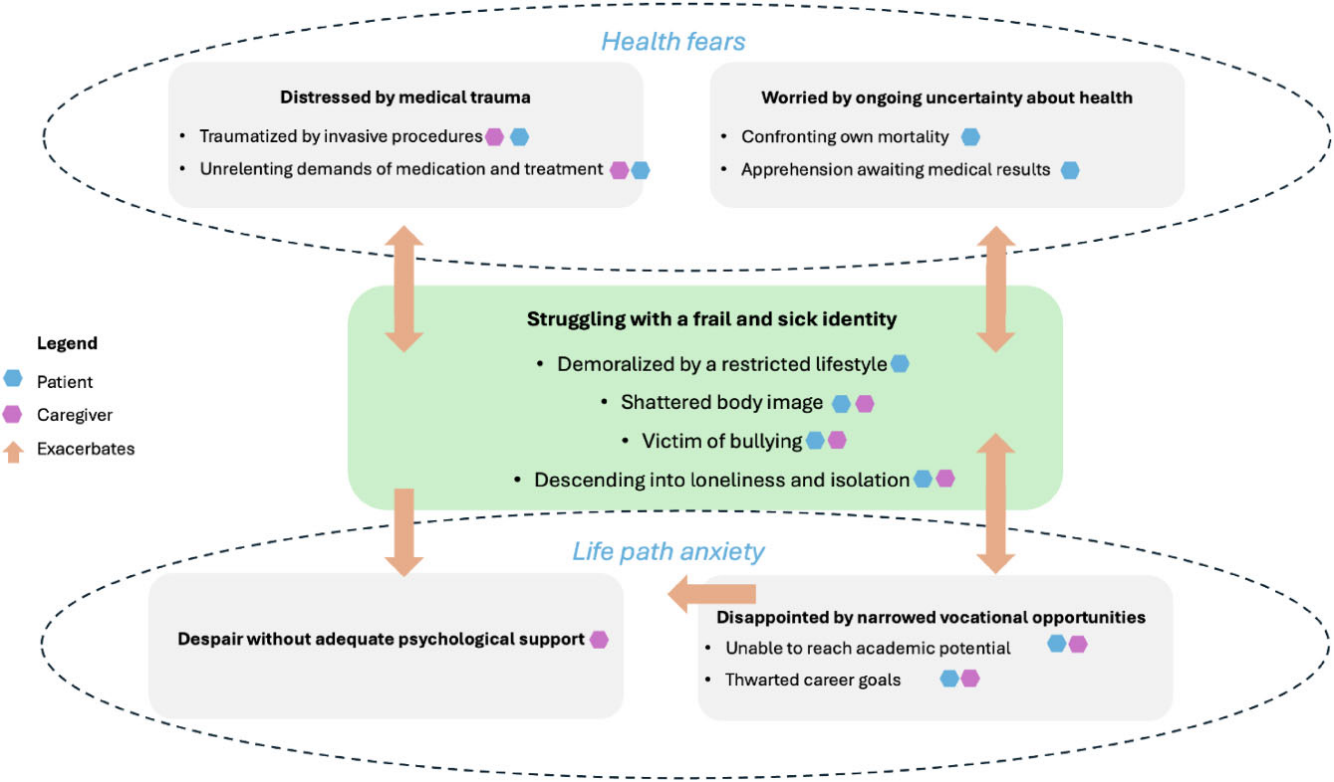
Thematic schema.

The characteristics of the participants (focus groups: *n* = 34 patients, 62 caregivers; Delphi survey: *n* = 72 patients, 132 caregivers; consensus workshops: *n* = 14 patients, *n* = 56 caregivers) are summarized in Table 1. Patients were aged from 8 to 21 years. Five themes were identified: struggling with a frail and sick identity, worried by ongoing uncertainty about health, disappointed by narrow vocational opportunities, distressed by medical trauma and despair without adequate psychological support. The respective sub-themes are described below. The conceptual links among the themes are depicted in Fig. 1. Illustrative quotes to support the themes are provided in Table 2.

**Table 1:** Participant characteristics.

Characteristic	Nominal group technique focus groups, *N* = 96^a^ [9], *n* (%)	Delphi survey, *N* = 204^a^ [15], *n* (%)	Consensus workshops, *N* = 70^a^ [16], *n* (%)
Participant group			
Patient	34 (35)	72 (35)	14 (20)
Caregiver/family member/friend	62 (65)	132 (65)	56 (80)
Gender			
Male	32 (33)	67 (33)	ns
Female	64 (67)	134 (66) 67	ns
Missing		3 (1)	ns
CKD stage^b^			
CKD^c^	42 (44)	59 (29)	ns
Dialysis	19 (20)	53 (26)	ns
Transplant	34 (35)	92 (45)	ns
Missing	1 (1)		
Patient's age^d^ (years)			Mean = 13 (SD 4.4)
8–18	30 (88)	49 (68)	ns
19–21	4 (12)	16 (22)	ns
Missing	0 (0)	7 (10)	ns
Country			
Australia	24 (25)	27 (13)	ns^h^
USA	23 (24)	69 (34)	ns^h^
Canada	13 (14)	17 (8)	ns^h^
UK	ns^e^	22 (11)	0
Singapore	0 (0)	21 (10)	0
India	ns^e^	17 (8)	0
New Zealand	ns^e^	15 (7)	0
Other	27^f^ (28)	16^g^ (8)	0
Missing	9 (9)		

a Numbers reported are for patients, caregivers and family members/friends. Health professionals also participated in these studies but their numbers are not reported here as their data were not included in the secondary analysis.

b CKD stage of patient or of the patient who the caregiver/family member is caring for.

c CKD, chronic kidney disease not requiring kidney replacement therapy.

d Age is reported in categories or as mean (SD), depending on what was available in the paper.

e India, England and New Zealand are also included in ‘Other’ for nominal group technique.

f Other includes up to 11 countries: England, Ethiopia, Fiji, India, Kenya, Mexico, New Zealand, Pakistan, Poland, Somalia and Vietnam.

g Other includes eight countries (in descending order of number of participants in round 1): France, Morocco, Myanmar, Malaysia, Pakistan, Philippines, Portugal and Saudi Arabia.

h Numbers for each country not reported but patients/caregivers came from USA, Australia and Canada.

*N* of patients, parents/family member or healthcare professional; ns, not stated; SD, standard deviation.

**Table 2:** Selected supporting quotations for each theme.

Struggling with a frail and sick identity
Demoralized by a restricted lifestyle
• ‘The pain. And the disability that it gives you as well. If you want to go to a theme park and you can't go on certain rides because of the restrictions on it’ (Patient)
• ‘It is really hard and you can't control yourself and have what you like’ (Patient)
• ‘And it's sort of like, because I have that nothing else, it's not really much enjoyment I guess’ (Patient)
Shattered body image
• ‘This was when I was self-conscious with myself, when I was taking the medication’ (Patient)
• ‘It is difficult for children, especially boys to be short in stature’ (Caregiver)
• ‘Certain drugs, namely steroids, cause side effects in the way you look and can make it even harder to deal with a diagnosis because you don't even look the same’ (Patient)
Victim of bullying
• ‘Or they shout it out to the whole school and then the whole school knows that you have a disability. And an easy target’ (Patient)
• ‘She doesn't like her teeth, because the medicine changed just how her teeth look, like they're slightly brown at the front, that's mainly because of her medication. And there was a girl who would tell her about her teeth and she's like, I know. Yeah, she doesn't like how her teeth look sometimes’ (Caregiver)
Descending into loneliness and isolation
• ‘If my child can't participate, she feels isolated and different to her peers’ (Caregiver)
• ‘Children who cannot keep up with their friends and peers are often left behind socially’ (Caregiver)
• ‘Feeling odd, left out, different’ (Patient)
Worried by ongoing uncertainty about health
Confronting own mortality
• ‘The impact this disease on the longevity or quality of our daughter's life is always in our thoughts’ (Caregiver)
• ‘When there is a possibility you could die it's hard to focus on much else’ (Patient)
• ‘My son worries, he thinks about it all the time. He doesn't think he'll live past 40, he'll tell us that’ (Caregiver)
Apprehension awaiting medical results
• ‘When I get bad results I get really bad anxiety. Because I don't know’ (Patient)
• ‘Can be stressful watching the creatinine levels fluctuate’ (Patient)
Disappointed by narrowed vocational opportunities
Unable to reach academic potential
• ‘This has been the biggest struggle. He has not been the same academically since transplant’ (Caregiver)
• ‘Achieving school potential is essential to growing up to be a productive and happy adult’ (Caregiver)
• ‘I went from making A's and B's to barely making C's. It would have been easier if I had some help from the hospital originally’ (Patient)
Thwarted career goals
• ‘Yeah I guess it's like if you have a job and you need a big operation and you have to take a lot of time away from work’ (Patient)
• ‘Has a major impact on self-management and concordance with treatment as child moves to adulthood. Also impact on educational attainment and future employment prospects’ (Caregiver)
Distressed by medical trauma
Traumatized by invasive procedures
• ‘The constant poking can cause panic attacks’ (Caregiver)
• ‘I think he had it harder because he absolutely hates needles and he has to have it so many times and he doesn't understand and he is crying all the time and it breaks my heart’ (Caregiver)
Unrelenting demands of medication and treatment
• ‘Well some of the hard things is always having to take tablets morning and night as well as drinking a lot of water during the day and having to go to the hospital a lot to see different doctors’ (Patient)
• ‘I want to travel the world. But having to deal with things like medications sometimes takes the fun out of it’ (Patient)
Despair without adequate psychological support
• ‘I think support. There's a lack of support for people living with kidney disease’ (Caregiver)
• ‘That's something I think the renal team within the hospital should even just look at, having a psychologist on board as part of their team’ (Caregiver)

### Struggling with a frail and sick identity

#### Demoralized by a restricted lifestyle

Patients and caregivers believed that lifestyle restrictions imposed by CKD limited the patient's ability to enjoy life, which impaired their mental health. The need to adhere to a strict diet and take medications prevented patients from feeling like ‘normal kids’, who felt they were constantly told you ‘can't do this or you'll end up getting sick’. Younger patients felt isolated and frustrated, particularly as they were unable to take part in school, sports and other social activities. Adolescents found it difficult being unable to ‘go out’ to social events and parties—‘When you are a teenager it stops you from experiencing things like drinking and stuff’ (patient), resulting in ‘isolation’ and ‘disappointment’.

#### Shattered body image

Symptoms of CKD and side effects from medications including ‘puffiness,’ ‘stained teeth’ and ‘stunted growth’ caused some patients to ‘hate themselves’ and become ‘depressed’. Patients felt ‘embarrassed’ when asked about medical devices such as gastronomy tubes. Some patients felt judged by other children because of their surgical scars—‘if you have a scar people see it and judge you’. Patients reported having an impaired body image, feeling rejected and isolated from their peers. Boys felt self-conscious about their short stature and being labelled as short—‘It is difficult for children, especially boys to be short in stature—it is often hard on their self-esteem’ (caregiver). Shorter stature was observed by caregivers to cause ‘sadness’ and ‘depression’ in their child. Some patients felt ‘sad and hopeless’ due to a sense of low self-worth and uncertainty about their future because of their illness. Kidney transplant recipients specifically attributed low self-esteem to the side effects of their medications. A caregiver expressed that their child saw themselves only as a ‘sick person and that's all he ever will be’.

#### Victim of bullying

Some patients felt ‘annoyed’ and had a diminished sense of self-esteem when peers ridiculed and laughed at them, particularly because of the appearance of medical devices—‘They made me show my (gastronomy tube) then one of the girls said you are yuck and started laughing at me for it’ (patient). Patients felt rejected and bullied by children due to their CKD—‘Or they shout it out to the whole school and then the whole school knows that you have a disability. And an easy target’. Patients reported instances of their ‘health information circulating’ around the school, which led to increased bullying, as one patient noted: ‘It's another way to be bullied. You tell someone (about the kidney disease), and they tell another person, and it goes everywhere, and they pick on you.’ Some caregivers observed how their child ‘suffered’ being told (by peers) that they were ‘faking it’ (illness), were ‘a wuss’ and there's ‘nothing wrong with you’.

#### Descending into loneliness and isolation

Caregivers reported their child felt ‘odd’ and ‘left out’ being unable to participate in activities such as team sports—‘football was hard enough for him because that's the whole team sport for a boy, that's something they really cling to’. Patients and caregivers were concerned that frequent hospitalization ‘disrupted the ability to form good friendships’ and caused patients to ‘lose friends’, which intensified isolation. Some worried they would never have a romantic relationship causing low confidence and impaired self-esteem—‘he's worried he will never have a girlfriend’ (caregiver) due to people wanting the ‘perfect person’ (patient).

### Worried by ongoing uncertainty about health

#### Confronting own mortality

Some young patients with kidney failure were fearful of death and anxious about their health—‘It scares us thinking of death’. Some patients felt stressed and fearful about surgery including for kidney transplantation due to complications including death—‘I was very afraid to do the operation and I was trying to persuade the doctors and my parents not to do it because I was super scared, what if I didn't wake up.’

#### Apprehension awaiting medical results

Patients felt nervous when awaiting medical test results, and found it ‘stressful’ when they saw, ‘fluctuations in renal levels’. Some caregivers of children receiving dialysis noted their child was ‘anxious’ about the unknown.

### Disappointed by narrow vocational opportunities

#### Unable to reach academic potential

Having to take absence from school due to illness and hospitalization exacerbated stress in patients because they struggled to perform academically—‘I'm falling behind, and if I fall behind, I feel as if I won't be able to catch up as easily’—and were ‘failing subjects’ (patients). Caregivers were concerned how poor academic performance could undermine their child's ‘psychological well-being’ and impair ‘self-esteem’ and ability to gain ‘independence’ in the future, further leading to limited opportunities in the future and low self-worth.

#### Thwarted career goals

Some patients felt anxious that their capacity and opportunities to find employment were diminished because of CKD as some jobs required them to be ‘physically fit’, or they would need to ‘take time off’ due to medical appointments. Some believed they had to be particularly careful in considering their career path to avoid putting their health at risk—‘provided my aim is Biomed, I'm not sure if I will be able to go in that area, the substances which are used may be potentially dangerous to my skin or my kidney. It really brings up worry in me’ (patient). Caregivers were also concerned about discrimination being a cause of low self-esteem and isolation in the workplace settings—‘nobody will employ my daughter’.

### Distressed by medical trauma

#### Traumatized by invasive procedures

Some patients felt ‘scared’, ‘anxious’ and ‘worried’ about medical procedures with some reporting having ‘panic attacks,’ ‘post-traumatic stress disorder’ and ‘psychological trauma’ from the ‘the pokes, the prods, and all the procedures’. Some caregivers reported their child's distress and ‘crying’ continued at home if they had to undergo a medical procedure—‘She loses the plot’, and ‘won't sleep all night because of blood test’. The anxiety because of treatment was perceived to cause emotional and behavioural challenges. A caregiver stated that when their child was about to undergo a transplant: ‘he got very angry and said he would rather have died... he just didn't want to be there’.

#### Unrelenting demands of medication and treatment

Having to take medications was ‘burdensome’ and ‘confusing’ for patients, which contributed to overwhelming ‘stress’. Many patients felt ‘exhausted’ by their treatment regimen and those on long term-medication wanted to cease taking medications. Patients receiving dialysis felt mentally exhausted by the frequent and ongoing hospitalizations, and worried about missing out on school. Some caregivers attributed ‘mood swings’, ‘stress’, ‘depression’ and ‘anxiety’ as side-effects of immunosuppression medications.

### Despair without adequate psychological support

Caregivers particularly in rural areas encountered difficulties accessing psychological support, with child-specific mental health services located ‘up to two hours away’. They did not believe that adult psychologists were appropriate: ‘I don't think an adult psychologist is the answer for a thirteen-year-old kid’ (caregiver). Some suggested the need for ‘psychologist within the renal team’ (caregiver). The lack of psychological support for their child led to desperation and worry in caregivers—‘If you’re happy to have the death of a ten-year-old on your hands, no worries, you all suck… this is at a point where she is saying I just want to die.’ Caregivers felt saddened and unable to help with their child's fear and depression.

## DISCUSSION

Children and adolescents with CKD struggled with a frail and sick identity, poor body image, lifestyle disruptions and restrictions, and bullying and judgement from others, which contributed to their sense of demoralization, loneliness and isolation. They felt anxious about their prognosis, medical results and having to confront their own mortality, particularly when they had to undergo surgery, which contributed to medical trauma. The treatment burden, including the relentless routine of dialysis and taking medications, also took a toll on their psychological state. Young patients with CKD and their caregivers were worried that having CKD would narrow academic and vocational opportunities. Caregivers unable to access psychological support for their child felt desperate and helpless about the severe psychological challenges they observed in their child with CKD, including suicide ideation.

Whilst patient and caregiver perspectives on mental health appeared common across demographic and clinical characteristics, some differences were noted based on age, CKD treatment stage and between patients and caregivers. Younger patients felt isolated and frustrated by the lifestyle constraints CKD had placed on their lives, particularly as they were unable to take part in school, sports and other social activities. Adolescents felt isolated from peers and were worried about narrowed opportunities in the future in terms of study, work and forming relationships because of the instability of their health. Patients receiving dialysis felt mentally exhausted by the frequent and ongoing hospitalizations, and worried about missing out on school. Patients appeared to focus more on the detrimental impacts on mental health that were related to body image concerns because of changes in appearance (e.g. swelling) and restrictions. Caregivers on the other hand felt helpless in managing their child's distress, anxiety and depression.

Prior studies have also found the demands of treatment, including frequent appointments, dialysis and hospitalization, dietary restrictions, school absenteeism and poor academic performance, contribute to poor mental health in young patients with CKD and their caregivers [2, 17–19]. Symptoms, side effects and complications, including swelling, have also been reported to cause young patients with CKD to feel abnormal and adversely affect their body image [2, 18]. Some felt different because they were ‘short’ in stature, which has also been documented in prior studies [19]. In previous work, young adults who had childhood CKD have also highlighted mental health struggles related to lifestyle limitations, limited vocational opportunities and a restricted social life [20]. Medical trauma resulting from needle-related procedures has also been found to be common in children and adolescents [21]. Our work has highlighted aspects of medical trauma that may be specific to and compounded in the paediatric CKD population given the need to undergo multiple invasive procedures including cannulation and surgery for vascular access and transplantation. Also, our findings emphasized distress in children with CKD related to awaiting test results and having to confront their own mortality, particularly if they required surgery.

We used a comprehensive multinational dataset including a diverse range of data sources. However, there are some potential limitations. This study was a secondary analysis of data from the SONG-Kids studies which focused on prioritizing outcomes for trials in paediatric nephrology, and therefore the questions in the primary studies were not explicitly focused on mental health. Furthermore, while this study was international, the participants were mostly from high-income English-speaking countries and therefore the transferability of our findings to other contexts may be limited. Of note, we did not identify any positive perspectives about mental health, for example in terms of coping and resilience.

There are several implications for practice that can be drawn from our findings. There is a need for clinicians to recognize, identify, validate and address the challenges related to restrictions on daily living, body image, self-esteem, coping with the side effects of medications and medical trauma. We suggest that care for children and adolescents with CKD involve a multidisciplinary team, including psychologists, psychiatrists and social workers, to screen, identify and address mental health issues in children and adolescents with CKD [6]. To ensure equity, barriers should be addressed so that support is readily accessible for all children and their families, particularly those living in rural and remote communities or experiencing socioeconomic disadvantage.

Various psychological interventions may be potentially effective in addressing the mental health challenges as identified in our study. Psychoeducation involves using educational tools to target stressors to support positive behaviours to maintain health [22–24], such as addressing ways to manage side effects to support treatment adherence. Cognitive behavioural therapy (CBT) is a goal-focussed technique that is used to alleviate emotional distress, and to support the establishment of positive cognitive patterns and behaviours. CBT may also be useful for addressing distress and pain, including those related to medical interventions [21]. A systematic review found that CBT delivered online among children with chronic pain may improve depressive symptoms, anxiety and sleep quality [25]. CBT has also been shown to potentially improve health-related quality of life and reduce depressive symptoms in adults with CKD [25]. Motivational interviewing is a counselling technique designed to enhance motivation and change in behavior [26]. A randomized trial in young people with chronic illnesses and anxiety found that motivational interviewing improved patient-clinician conversations and decreased anxiety [26].

Health coaching can be used to address concerns about academic and vocational goals, and coping with chronic illnesses, and has been shown to help adult patients with kidney failure achieve desired goals, better capacity for self-management and overall quality of life [27]. Effective health coaching supports the emotional wellbeing of a patient by providing a health coach who helps build confidence and coping skills to manage the mental health challenges of a chronic illness [28]. Health coaches achieve this through communication, listening and providing a continuity of care [28]. Future work should focus on co-designing of interventions to support mental health in young people with CKD, in collaboration with patients and their caregivers, evaluating their effectiveness and informing real-world implementation.

We also suggested that multidisciplinary support, including from physicians, psychologists and social workers, as well as from community-based organizations (i.e. schools) may help to support the mental health of children with CKD.

Children and adolescents experience severe mental strain from the challenges of living with CKD. Providing mental health support to patients and caregivers affected by CKD is likely to improve well-being and quality of life, assist patients and families in dealing with stress and trauma, and provide a positive patient experience of kidney care.

## Supplementary Material

sfaf067_Supplemental_File

## Data Availability

The data underlying this article are available in the article and in its online supplementary material.
