# Supplementary material for: Patient and caregiver perspectives on mental health in children and adolescents with chronic kidney disease
Source: Clin Kidney J. 2025 Mar 8;18(4):sfaf067. doi: 10.1093/ckj/sfaf067 (PMC11986815; doi:10.1093/ckj/sfaf067)
Supplement: sfaf067_Supplemental_File [file sfaf067_supplemental_file.docx]

**Supplementary material**

**Supplementary File 1.**

List of institution review boards and participating institutions that provided ethics approval for SONG-Kids studies.

- The Sydney Children’s Hospital Network, Sydney, New South Wales, Australia (HREC/16/SCHN/70)
- Royal Children’s Hospital, Melbourne, Victoria, Australia (HREC/16/SCHN/70)
- Monash Health, Melbourne, Victoria Australia (HREC/16/SCHN/70)
- The Alberta Children’s Hospital, Calgary, Alberta, Canada (REB16-0255)
- British Columbia Children’s Hospital, Vancouver, British Columbia, Canada (HREC/16/SCHN/70)
- Baylor College of Medicine, Houston, Texas, United States (H-39032)
- The University of Sydney, Sydney, New South Wales, Australia (2015/228)
